# Realtime monitoring of internal morphology of gel samples during drying process by X-ray computing tomography image using synchrotron radiation

**DOI:** 10.1038/s41598-025-18840-y

**Published:** 2025-10-07

**Authors:** Daitaro Ishikawa, Yuzuka Ito, Masafumi Hidaka, Jiamin Yang, Kanna Yamada, Mio Takiguchi, Motofumi Otomo, Tomoyuki Fujii

**Affiliations:** 1 Graduate School of Agricultural Science, Tohoku University, 468-1 Aramaki Aza Aoba, Aoba-ku, Sendai, Miyagi 980-8572 Japan; 2 Hatakenaka Seimen, Japan Industry, 4-11 Otemachi, Shiroishi, Miyagi 989-0276 Japan

**Keywords:** X-ray computed tomographic image, Structural image analysis, Drying process, Synchrotron

## Abstract

This study was conducted to evaluate the change in the internal morphology of a wheat-based gel sample subjected to a drying process using X-ray computed tomography (CT) imaging with synchrotron radiation. To monitor the process in real time, the instrument was equipped with a developed drying system. The water contents of the sample decreased from around 0.42 to 0.1 g-water/g-dry solid and the falling rate drying was observed in the 0–180 min of drying period. The mechanical properties of the gel sample, represented by the Young’s modulus, increased exponentially during the drying period. Thus, when subjected to low-water-content conditions, the hardness of the gel progressed sufficiently. In the X-ray CT images, small voids were generated uniformly in the sample during the early drying process, and the total number of voids and their volume also increased. Finally, they formed a band-like structure at the center of the sample. The total number of voids decreased at a rate of increase with the confluence of voids, and eventually reached a plateau. These results suggest that voids may be connected and cracked during drying. It was found that the drying process of a gel sample in this study proceeds in two phases: a void growth phase up to 60–80 min and thereafter, a crack generation phase. Consequently, the internal morphology of the gel was visualized using synchrotron-based X-ray CT imaging in real time, and it was demonstrated that the method is very effective for monitoring a gel during the drying process.

## Introduction

The structure of gel samples in the wet state is generally fragile because of the presence of uncrosslinked hydroxyl groups and other components^1–4^. Therefore, many gel samples are formed in a high-water-content state and are used in a stable glassy state after the drying process^1–4^. When purifying gels using gelation techniques and polymer synthesis processes, a large amount of water or alcohol must be evaporated after gelation^5,6^. At this time, cracks at the nanoscale are likely to occur inside the gels, and the larger the sample size, the more difficult it is to dry the gel as a monolith. Because the drying process is used in a wide range of fields such as pharmaceuticals, materials, and food to improve product stability and impart functional properties^7–9^, the internal breakdown of gel materials is one of the greatest concerns because it is directly related to product defects.

Aerogels are usually synthesized by supercritical drying through diligent solvent exchange and drying procedures^5^, In the supercritical drying method, since the phase boundary between liquid and gas is not crossed, capillary forces do not occur and the gel structure can be dried in a perfect state without being destroyed. However, in ambient pressure drying, which is an alternative approach for aerogels, and the vacuum drying method, which is commonly performed for practical samples, the evaporation of liquid from the gel network creates compressive stress in the solid phase, resulting in network contraction. If the gel backbone is flexible, shrinkage occurs; if it is relatively firm, fracture occurs^9^. The mechanical properties of the gels decrease when cracks occur. Because cracks cannot be identified from the surface, the issue is usually evaluated by numerical analysis and mechanical testing during the process^9–11^. However, it is necessary to evaluate not only the mechanical properties but also the degree of cracking to control the gel materials. Therefore, the development of methods that allow the non-destructive, real-time assessment of internal anomalies is critical for material control and improvement.

X-ray computed tomography (CT) is one of the most effective approaches to non-destructive observation of three-dimensional material structures at the nanoscale, so that it can be applied to not only medical and heavy industrial fields (relatively low resolution), but also to various applications such as engineering, food, and biological sciences with higher resolution^12–16^. For CT imaging, X-rays are used to create and record a series of radiographs (2D) from different sample angles. The internal 3D microstructure was reconstructed using a specific reconstruction algorithm^17^.

Regarding the internal state of the gel, Ukoji et al. reported the effect of different mechanical treatments on the physical properties of gel material powder by investigating the internal structure using X-ray CT images and the texture of gel foods^18^. Roh et al. reported that the inner structure of the noodles was visualized by micro-CT analysis based on density differences, and their degree of processing was successfully obtained based on three-dimensional images. In addition, three water populations were clearly identified in the noodle sample, combined with proton NMR analysis^19^. In the gel drying process, many evaluations have been conducted using porosity as a parameter because changes in porosity affect physical properties^18,20^. Yasuda et al. and Hosoda et al. reported that the void structure of dried noodles can be either zonally or randomly distributed, depending on the mechanical treatment method^21,22^. Suzuki et al. developed a method for determining the distribution of cracking damage in the internal structure of concrete samples by applying the X-ray CT test. Although the experiment was not performed on gel samples, it is interesting to note that cracking damage corresponds to the porosity^23^. However, to the best of our knowledge, changes in the internal morphology of soft materials such as food gel samples during the drying process have not yet been clarified. Real-time understanding of the internal conditions during the drying process has not been conducted thus far because there are mechanical constraints for real-time monitoring of the three-dimensional properties of an object with high spatial resolution. As Villanova et al. and Vladmir et al. have stated, synchrotron-based X-ray CT is currently the only imaging technique capable of capturing the dynamic evolution of multiphase systems, such as the drying process, with µm-scale spatial and second-order temporal resolutions^24,25^.

Thus, the aim of this study was to develop a non-destructive method for quantifying changes in the internal morphology of a gel sample during the drying process. Wheat-based gels were used in this study. The main component of a wheat-based gel sample, udon noodles, is wheat flour. The starch and gluten contained in wheat flour combine with water during the kneading process; when heated, they form a strong network structure. Gluten, in particular, is extremely important for the binding of proteins to create an elastic network. The “firmness” that is unique to the udon noodle is precisely the elasticity that this network structure provides. The unique chewiness that you feel when you bite into it is due to the properties of the gel. Thus, the udon noodle matches the definition of a gel very well in terms of its structure and properties. In other words, the starch and gluten in wheat flour form a three-dimensional network structure, and since it has solid elasticity, it can be said that the udon noodle is classified as a gel. In addition to udon noodles, there are many other gels around us as well. For example, jelly, agar, rubber, and PVA gels are also types of gel. These polymers also formed a network structure. Therefore, the approach implemented in this study can be expected to be generally used for many gel objects, as well as food materials.

To enable real-time measurements in this study, a drying apparatus that can be connected to an X-ray CT system was created. X-ray CT images were obtained continuously during the drying process. Elucidation of the void structure and generation of cracks in the gel sample during the drying process was performed by structural imaging analysis of the CT image.

## Results and discussion

### Moisture contents and Young’s modulus in bending of gel samples

**Fig. 1:**
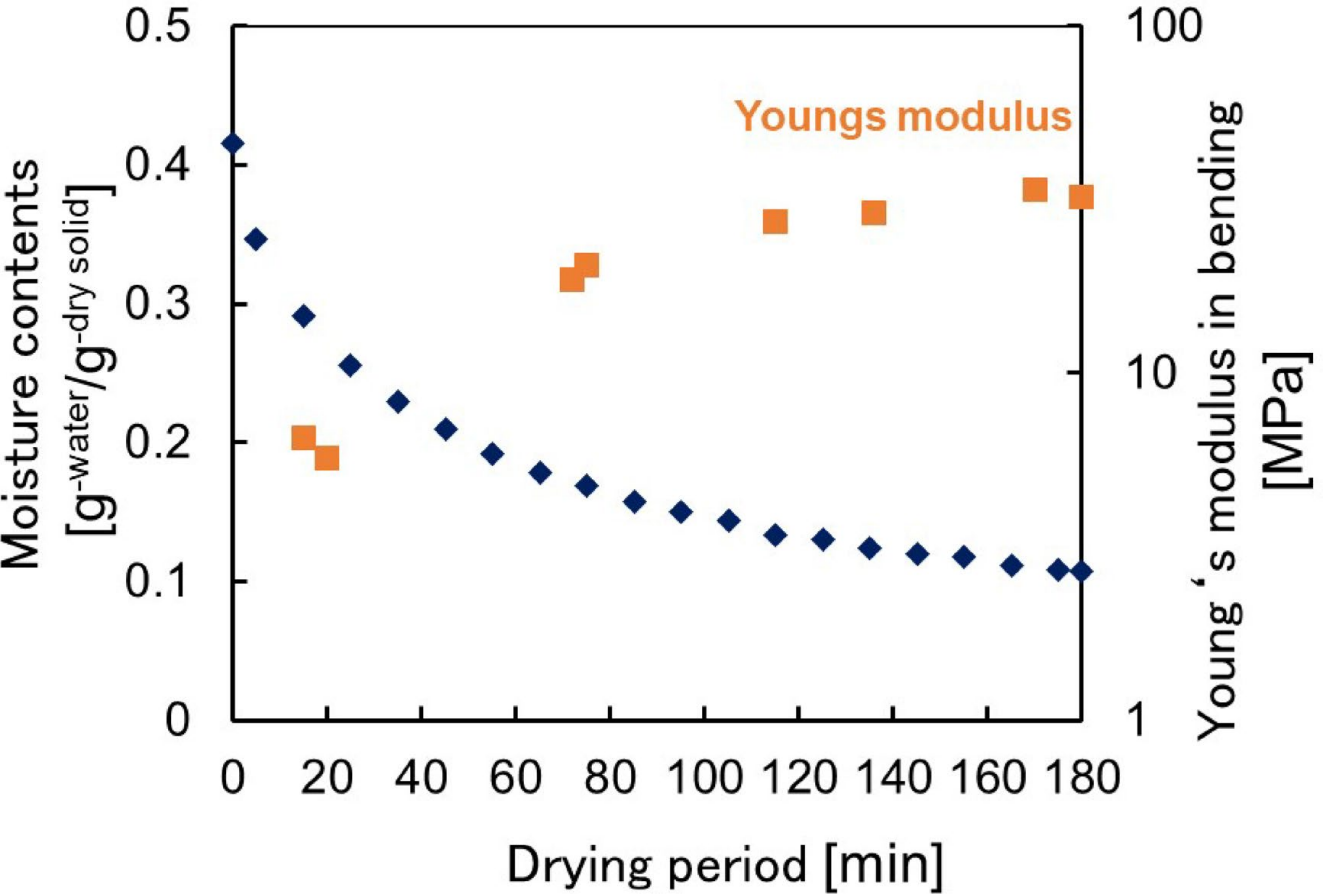
The course of moisture content during the drying process and the Young’s modulus in bending at each moisture contents.

The course of the moisture content during the drying process and the Young’s modulus in bending at each moisture content are shown in Fig. 1. The Young’s modulus was plotted as representative values corresponding to each moisture content. Moisture content decreased monotonically during the 0–180 min. The moisture content changed from around 0.4 to around 0.1 g-water/g-d. s, during the drying period. The course of moisture content in this sample did not have a constant rate drying period, confirming that it was reduced-rate drying. The Young’s modulus in bending increased to 60 min and mostly reached a plateau. A correspondence between the moisture content and Young’s modulus was observed, with a high and constant Young’s modulus in the dry cycle. In other words, at low moisture content, the gel hardened.

### Distribution analysis of voids in the gel samples

**Fig. 2:**
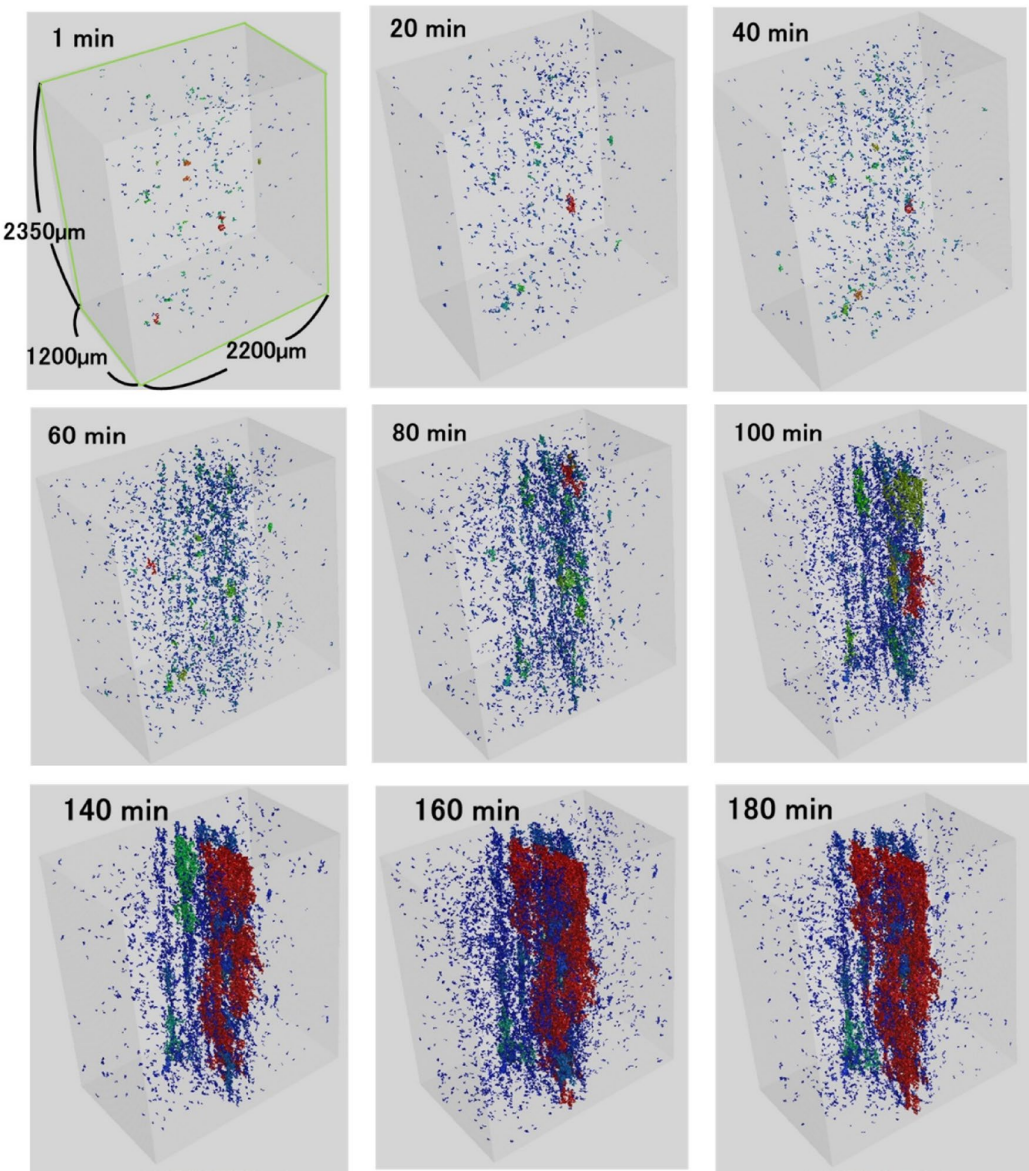
The course of three-dimensional X ray CT image of a gel sample during drying process in the 0–180 min.

Three-dimensional images of the dried samples are shown in Fig. 2. In the early stage of drying, a small void appeared, and the number of voids increased gradually. Compared to the initial stage, small large voids were also observed to occur slightly at this stage.

The number of voids increased in the 40–80 min period, and the void size was also larger than that in the early stage owing to void growth. Interestingly, the voids grew from the center of the gel during the drying period. Yamada et al. 2023 and Hosoda et al. reported that the pores were belt-like structures in starch-based gel samples^21,22^. They also suggested that the distribution could be uniform or zonal, depending on the sample conditioning method. In our study, we tracked the porosity during the drying process and found, for the first time, that fibrous porosity occurs inside the gel matrix depending on the degree of drying.

**Fig. 3:**
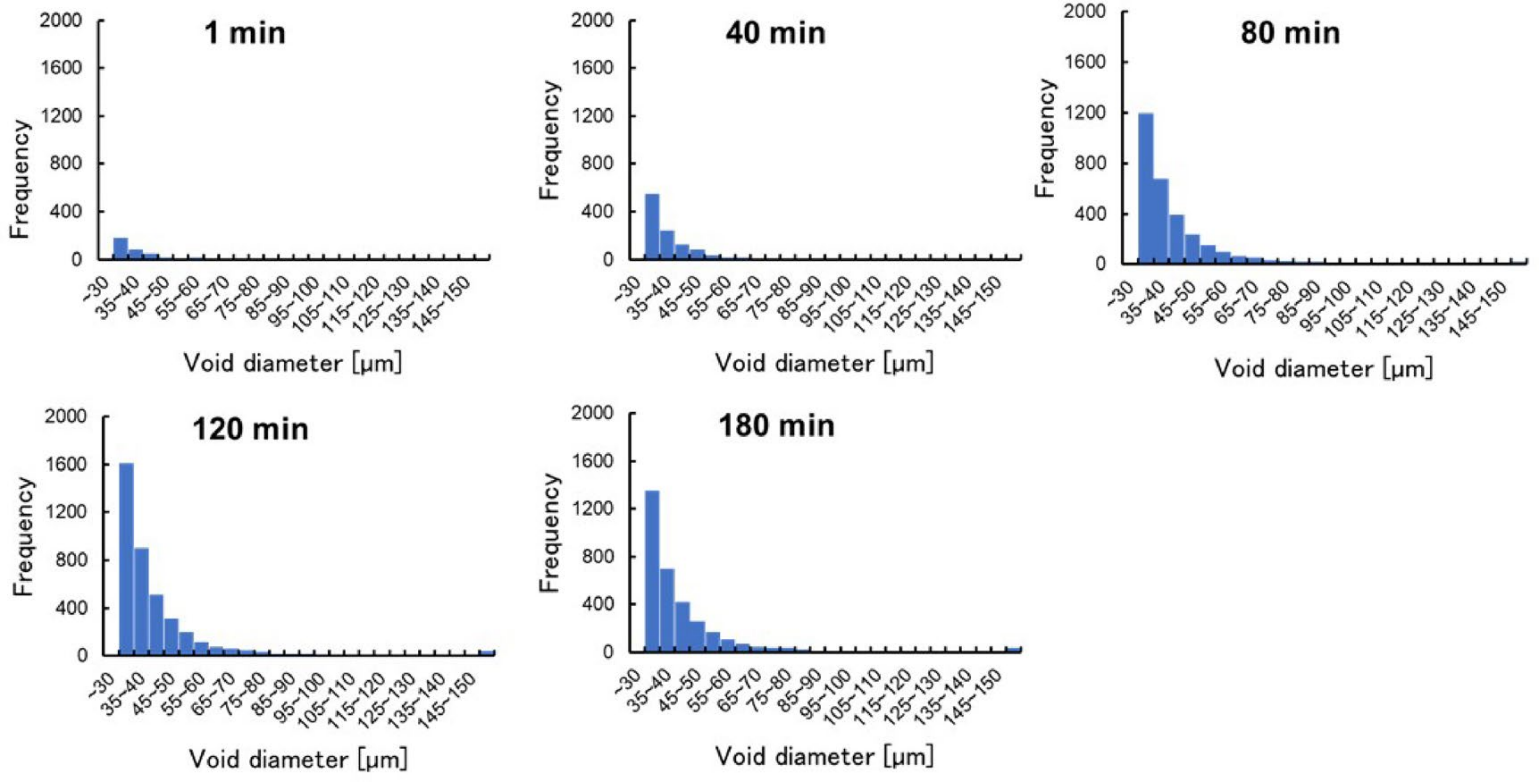
Change in distribution of void diameter in a gel sample during drying process in the 0–180 min.

The size of the voids increased even further as the voids connected. After 100 min of drying, the void size increased, indicating that internal cracking may have occurred. Figure 3 shows the change in the distribution of void diameters calculated from the CT images with elapsed time during the drying process. Throughout the drying period, the void diameters were approximately normal. It was found that the percentage of large voids increased as drying progressed. Furthermore, the number of voids decreased after 120 min.

**Fig. 4:**
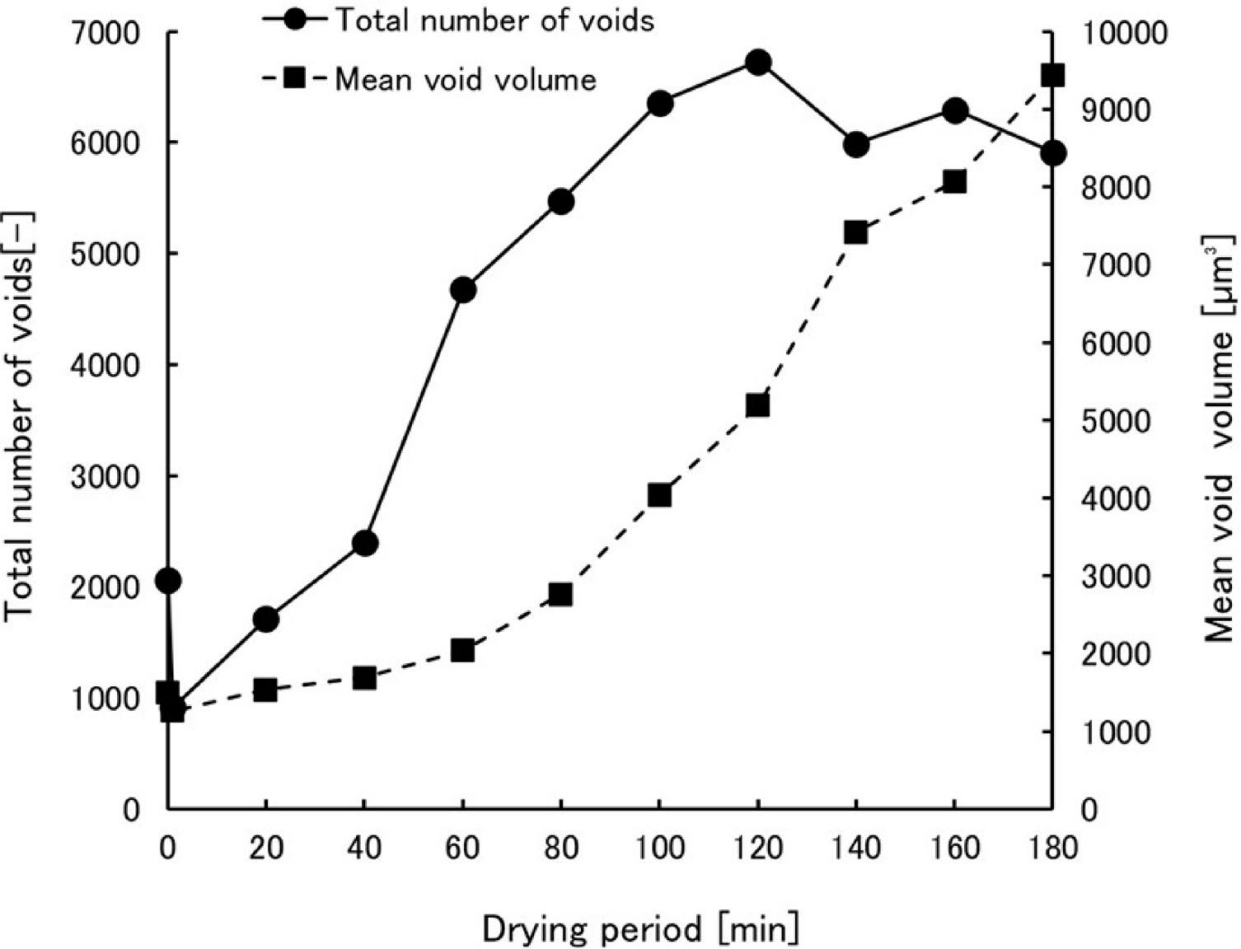
Course of the total number of voids and the mean void volume in a gel sample during drying process in the 0–180 min.

The course of the total number of pores and the mean pore volume during the drying process are shown in Fig. 4. The mean void volume changed slightly in the 0–60 min and it significantly increased to 9500 µm^3^ in the 60–180 min drying process. In the period up to 60–80 min, as shown in Fig. 1, an increase in the Young’s modulus in bending suggests that hardening was in progress. During this period, as shown in Fig. 4, the mean void volume changed slightly. Subsequently, the volume increased with the generation of cracks in the sample. It is very likely that the behavior of the mean pore volume corresponds to the physical properties. The total number of voids increased to approximately 6800 in the 0–120 min and subsequently decreased to 6000. The rate of increase in the total number of voids decreased after approximately 60 min. They then levelled off or started to decrease around 120 min. Therefore, the total number of voids indicates the possibility that the cracks generated in the sample were also counted as voids. It is likely that the total number of voids did not change significantly owing to the merging of cracks and voids and counting them as large voids though the voids were increasing in that period. Consequently, the drying process of the gel sample in this study was divided into a void formation phase up to 60–80 min, in which the number of voids increased significantly and the volume increased slightly, and a crack generation phase, in which cracks were formed, the volume increased significantly, and the rate of increase in the total number of voids decreased.

With respect to the drying of gel materials, it is believed that the drying progression is characterized by cracking at the surface, with a decreasing moisture content at the periphery and a high moisture content at the center^2^. However, previous studies have not sufficiently understood the state of the voids during the drying process. In this study, it was observed that if voids were internalized and their effect was not small, internal cracks were generated by the growth of voids in the center and cracks between voids, even if the water content was low at the surface and high at the center. It was strongly suggested that a poor understanding of dissolved air solubility and growth of voids in the drying process of hydrogel materials may result in products with weak strength owing to internal cracks that are not observed on the outside of the gel.

Overall, in this study, samples in the drying process were continuously observed by synchrotron-based CT images in the same field of view using the developed drying apparatus. Consequently, a clear distinction was successfully made between void growth and crack generation. Furthermore, the increase in voids in the falling-rate drying period from to 60–80 min onward was attributed to cracks. These results provide an attractive new monitoring method for real-time, nondestructive evaluation of changes in the internal morphology of materials in multiphase systems.

## Materials and methods

### Sample preparation

A wheat-based udon noodle (Hatakenaka Seimen Co., Ltd., Japan) was used as the gel sample. The sample mainly consisted of 9.8% protein, 1.2% lipids, and 76% carbohydrates. To produce a gel, wheat flour and sodium chloride were kneaded with approximately 30 wt% water for several minutes. The gel sample was rested for a while, and then a sheet was prepared by passing it through the roll to obtain a sheet of approximately 2 mm thickness. The sheets were cut to 3 mm each and allowed to stand until the moisture content was approximately 0.42 g-water/g-dry solid.

A commercially available wheat-based udon noodle (Hatakenaka Seimen Co., Ltd., Japan) was used as the gel sample. The composition of this sample consisted of approximately 9.8% protein, 1.2% lipids, and 76% carbohydrates (w/w, based on total solids), which is representative of traditional Japanese udon formulations. The gel was prepared by thoroughly mixing wheat flour with sodium chloride and water to achieve a total water content of approximately 30 wt%. The mixture was kneaded for several minutes using a mixer to ensure uniform hydration and to promote the development of a continuous gluten network, which serves as the primary structural framework of the gel. After kneading, the sample was rested at ambient temperature for a predetermined period to allow moisture equilibration and stabilization of the gluten structure. The rested sample was then processed into a sheet of approximately 2.0 mm thickness using a roller. The resulting sheet was cut into uniform strips with a width of 3.0 mm. The cut samples were placed on a perforated tray and allowed to stand under 25 °C until the moisture content reached 0.42 g-water/g-dry solid, as determined gravimetrically. This preparation protocol was designed to yield homogeneous gel samples with reproducible physicochemical and mechanical properties.

## Methods

### Drying process

**Fig. 5:**
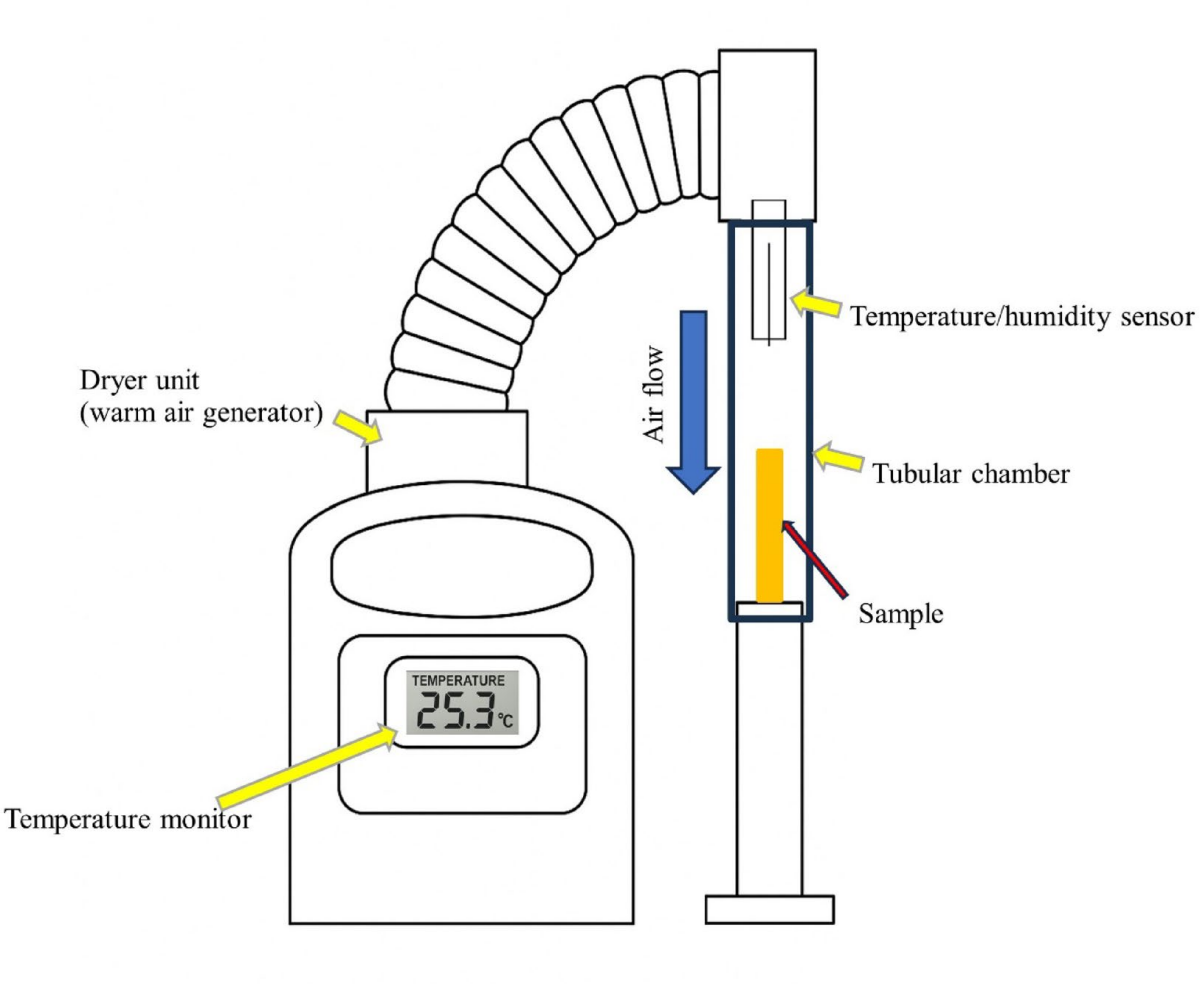
schematic diagram of the developed drying system.

The developed flow-drying system was used in this study. As shown in Fig. 5, the flow-drying system consisted of a tubular chamber, dryer unit, and temperature/humidity sensor. The samples were placed perpendicular to each other in the chamber. The drying process was continued for 180 min, and warm humified air flowed from the upper part of the chamber from the dryer unit. The flow speed of the air was 1.89 m/s, and the sample chamber was maintained at a temperature of 50 °C and a relative humidity of 22%. The weight of the chamber was measured intermittently using a weight scale during the drying period. The moisture content was quantified from the amount of weight loss at each time point.

### Mechanical properties

To investigate the mechanical properties of samples with different moisture contents, water activity control was performed in small desiccators filled with saturated salts for a water activity range of 0.11–0.98 of water activity. Each sample was stored in a desiccator until equilibrium was reached at 50℃. Finally, samples with nine moisture content levels were prepared.

Mechanical measurements were performed using a creep meter (model RE2-3305 s; Yamaden Co., Japan) equipped with a three-point bending fixture. In this setup, each sample was placed horizontally across two parallel beam supports, with a fixed span length of 16 mm between supports. The bending load was applied from above using a wedge-shaped plunger with a thickness of 5 mm and a width of 90 mm, ensuring uniform contact across the sample’s width. The plunger was driven downward at a constant crosshead speed of 0.1 mm/s to minimize dynamic effects and to obtain quasi-static mechanical response data. The Young’s modulus in bending (σ/ε) was calculated from the stress–strain curve using the following equation:

$$\delta {/}\varepsilon {}={\text{PL}}^{3} {/}4{\text{bh}}^{3} {\text{w}}$$

where δ and ε are the stress and strain in bending, respectively; P is the load [N]; b is the width of the sample [mm]; h is the height of the sample [mm]; w is the deformation [mm]; and L is the length between two separated fixtures (16 mm). Measurements were conducted in duplicate for each water activity condition.

### Measurement of CT images

**Fig. 6:**
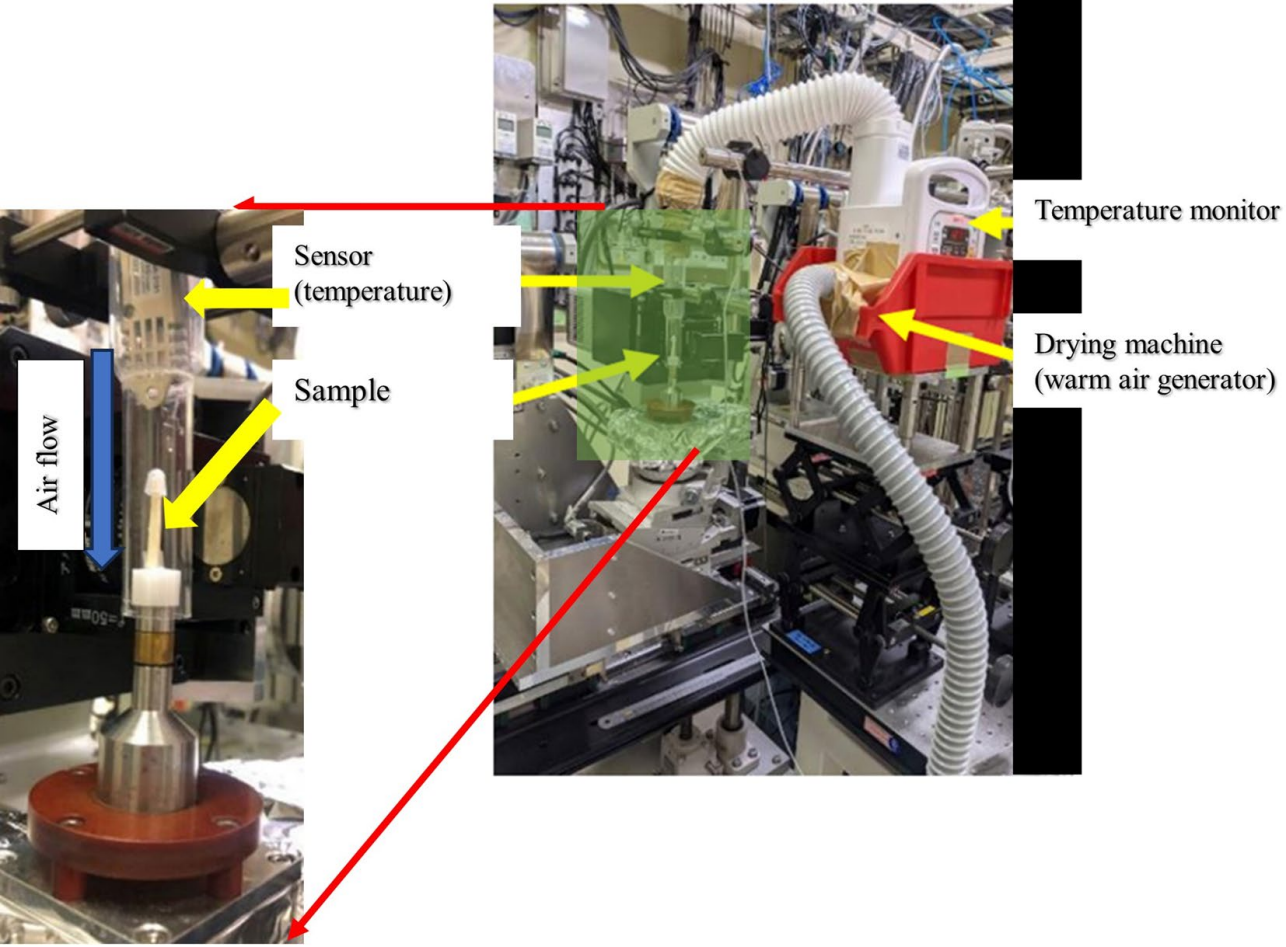
The schematic photo of the sample stage of X ray CT instrument equipped with the developed drying system (right) and a scale upped view of the sample chamber (left).

X-ray computed tomography (CT) of the sample was performed with BL14B2 of SPring-8 at 12.4 keV (≒1Å). To perform X-ray CT measurements in real time during the drying process, the flow system shown in Fig. 6 was placed on the X-ray CT instrument. The sample was held vertically in a sample holder of BL14B2, as shown in Fig. 6. The distance between the sample and the detector was 30 mm and the exposure time was 250 ms. A support rod was placed behind the sample to keep it vertical. Air at a temperature of 50 °C and relative humidity of 22% %constantly flowed from above to the sample in parallel for 0–180 min. The three-dimensional composition of the image was constructed using a beamline. The data were acquired in 1600 Tiff format images with a resolution of 2.93 μm/px. Each pixel had a 32-bit gradation. In order to improve the signal-to-noise ratio, averaging binning processing was performed with 4 × 4 × 4 voxels and analyzed as image data with a resolution of 11.72 μm/px.

### Structural image analysis

The part of sample was identified from the CT image, and the analyzed volume of the sample was 1200 × 2200 × 2350 µm. To reduce the noise, median filter and CT artifact reduction filter were applied prior to image analysis. In general, the CT images were expected to contain CT noise. In this study, the maximum value of CT noise was approximately φ11-12 µm based on the distribution of voids. Assuming that the noise is Gaussian distribution, it is considered to contain almost no noise if it is larger than 25 µm of a void size. In this study, thus the voids bigger than φ25 µm was extracted in a CT image. The total number of void and the mean void volume were investigated from each image. The structural image analysis was performed by VG studio MAX (Volume Graphics Co., Ltd.).

## Data Availability

The data underlying this article will be shared upon reasonable request to the corresponding author.
