# Correction: Realtime monitoring of internal morphology of gel samples during drying process by X-ray computing tomography image using synchrotron radiation

**DOI:** 10.1038/s41598-025-27958-y

**Published:** 2025-11-11

**Authors:** Daitaro Ishikawa, Yuzuka Ito, Masafumi Hidaka, Jiamin Yang, Kanna Yamada, Mio Takiguchi, Motofumi Otomo, Tomoyuki Fujii

**Affiliations:** 1 Graduate School of Agricultural Science, Tohoku University, 468-1 Aramaki Aza Aoba, Aoba-ku, Sendai, Miyagi 980-8572 Japan; 2 Hatakenaka Seimen, Japan Industry, 4-11 Otemachi, Shiroishi, Miyagi 989-0276 Japan

Correction to: *Scientific Reports* 10.1038/s41598-025-18840-y, published online 07 October 2025

The original version of this Article contained a typographical error in Equation 1, where “=” was omitted.

$$\delta {/}\varepsilon {}{\text{PL}}^{3} {/}4{\text{bh}}^{3} {\text{w}}$$

now reads:

$$\delta {/}\varepsilon {}={\text{PL}}^{3} {/}4{\text{bh}}^{3} {\text{w}}$$

The original Article has been corrected.

